# Transplantation of modified human bone marrow‐derived stromal cells affords therapeutic effects on cerebral ischemia in rats

**DOI:** 10.1111/cns.13947

**Published:** 2022-08-24

**Authors:** Satoshi Kawauchi, Takao Yasuhara, Kyohei Kin, Satoru Yabuno, Chiaki Sugahara, Takayuki Nagase, Kakeru Hosomoto, Yosuke Okazaki, Yousuke Tomita, Michiari Umakoshi, Tatsuya Sasaki, Masahiro Kameda, Cesario V. Borlongan, Isao Date

**Affiliations:** ^1^ Department of Neurological Surgery Okayama University Graduate School of Medicine, Dentistry and Pharmaceutical Sciences Okayama Japan; ^2^ Department of Psychiatry and Behavioral Neurobiology University of Alabama at Birmingham Birmingham Alabama USA; ^3^ Department of Neurosurgery Osaka Medical College Osaka Japan; ^4^ Department of Neurosurgery and Brain Repair, Center of Excellence for Aging and Brain Repair University of South Florida Tampa Florida USA

**Keywords:** bone marrow stromal cells, cerebral infarction, encapsulated cell transplantation, middle cerebral artery occlusion model, neurogenesis

## Abstract

**Aims:**

SB623 cells are human bone marrow stromal cells transfected with Notch1 intracellular domain. In this study, we examined potential regenerative mechanisms underlying stereotaxic transplantation of SB623 cells in rats with experimental acute ischemic stroke.

**Methods:**

We prepared control group, empty capsule (EC) group, SB623 cell group (SB623), and encapsulated SB623 cell (eSB623) group. Transient middle cerebral artery occlusion (MCAO) was performed on day 0, and 24 h after MCAO, stroke rats received transplantation into the envisioned ischemic penumbra. Modified neurological severity score (mNSS) was evaluated, and histological evaluations were performed.

**Results:**

In the mNSS, SB623 and eSB623 groups showed significant improvement compared to the other groups. Histological analysis revealed that the infarction area in SB623 and eSB623 groups was reduced. In the eSB623 group, robust cell viability and neurogenesis were detected in the subventricular zone that increased significantly compared to all other groups.

**Conclusion:**

SB623 cells with or without encapsulation showed therapeutic effects on ischemic stroke. Encapsulated SB623 cells showed enhanced neurogenesis and increased viability inside the capsules. This study reveals the mechanism of secretory function of transplanted SB623 cells, but not cell–cell interaction as primarily mediating the cells' functional benefits in ischemic stroke.

## Graphical abstract

This study demonstrated SB623 cells with or without encapsulation exerted therapeutic effects on acute ischemic stroke model of rats. Encapsulated SB623 cells enhanced neurogenesis and survived longer than unencapsulated SB623 cells.

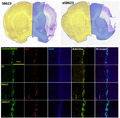

## 1 INTRODUCTION

Ischemic stroke is a leading cause of long‐term disability, representing one of the most serious health problems in the world.^1^, ^2^ Although there have been various innovative therapies for ischemic stroke, such as rt‐PA and mechanical thrombectomy, many patients still suffer from this disorder.^3^ Cell transplantation treatment has emerged as an experimental treatment for ischemic stroke.^4^, ^5^ In particular, bone marrow stromal cells (BMSCs) transplantation has been demonstrated as safe and effective for experimental ischemic stroke.^6^ BMSCs possessed cell regenerative features including their capacity for cell replacement and secretion of neurotrophic factors^7^, ^8^, ^9^ with the latter by‐stander effects of BMSCs gaining more compelling laboratory evidence than the former mechanism of cell differentiation into neurons.^10^ However, the specific mechanism mediating SB623 cells' therapeutic effects still remains uncertain.

SB623 cells are modified human BMSCs transfected transiently with a vector encoding the human Notch1 intracellular domain (NICD).^11^, ^12^ Transplantation of rat NICD transfected cells exerted more functional benefits in stroke rats than transplantation of untransfected BMSCs.^13^ Interestingly, SB623 cells secrete higher levels of various neurotrophic factors like IL‐6, IL‐8, FGF1, FGF2, and MCP‐1 than those of BMSCs.^14^, ^15^, ^16^ The enhanced supply of these trophic factors is considered a key to strong therapeutic effects of SB623, compared to control BMSCs. Intracerebral transplantation of SB623 cells showed promising results in clinical trials, despite a clear understanding of the cells' mechanism of action.^17^, ^18^, ^19^ Cognizant of the cell replacement and by‐stander effects of BMSCs^7^, ^10^, optimizing the cell delivery of SB623 cells to achieve such regenerative mechanisms stands as an important factor to improve the efficacy/safety of cell transplantation therapy. Various cell delivery routes, such as venous or arterial injection and direct injection to the brain, have been examined.^20^, ^21^ We previously reported that encapsulation of different cell lines promotes functional recovery through the secretion of neurotrophic factors. Our past studies showed encapsulation of BMSCs enhanced their cell regenerative features.^22^ Most xenogeneic or allogeneic cells transplanted directly into rodent's brains survive for only a few days or weeks because they are attacked by the host's immunoreaction.^22^, ^23^, ^24^, ^25^, ^26^ In contrast, encapsulated cells survive longer because the capsule protects the cells from hosts' immunoreaction, allowing the grafted cells to continue to secrete neurotrophic factors.^22^, ^23^, ^24^, ^25^, ^26^ In this study, we tested a two‐pronged hypothesis; first, that encapsulation would allow SB623 cells to survive more robustly when directly transplanted into brains of stroke rats; second, with encapsulation preventing cell‐to‐cell contact between the host and the grafted cells, we would be able to ascribe any therapeutic effects of SB623 cells to the by‐stander mechanism, i.e., secretion of trophic factors.

## 2 METHODS

### 2.1 Animals

Adult male Wistar rats (Charles River Laboratories Japan, Inc., Yokohama, Japan), weighing 280 to 320 g at the beginning of the experiment, were used in this study. They were group‐housed with two animals per cage in the temperature and humidity‐controlled room, maintained on a 12 h light/dark cycle, with free access to food and water.

**FIGURE 1:**
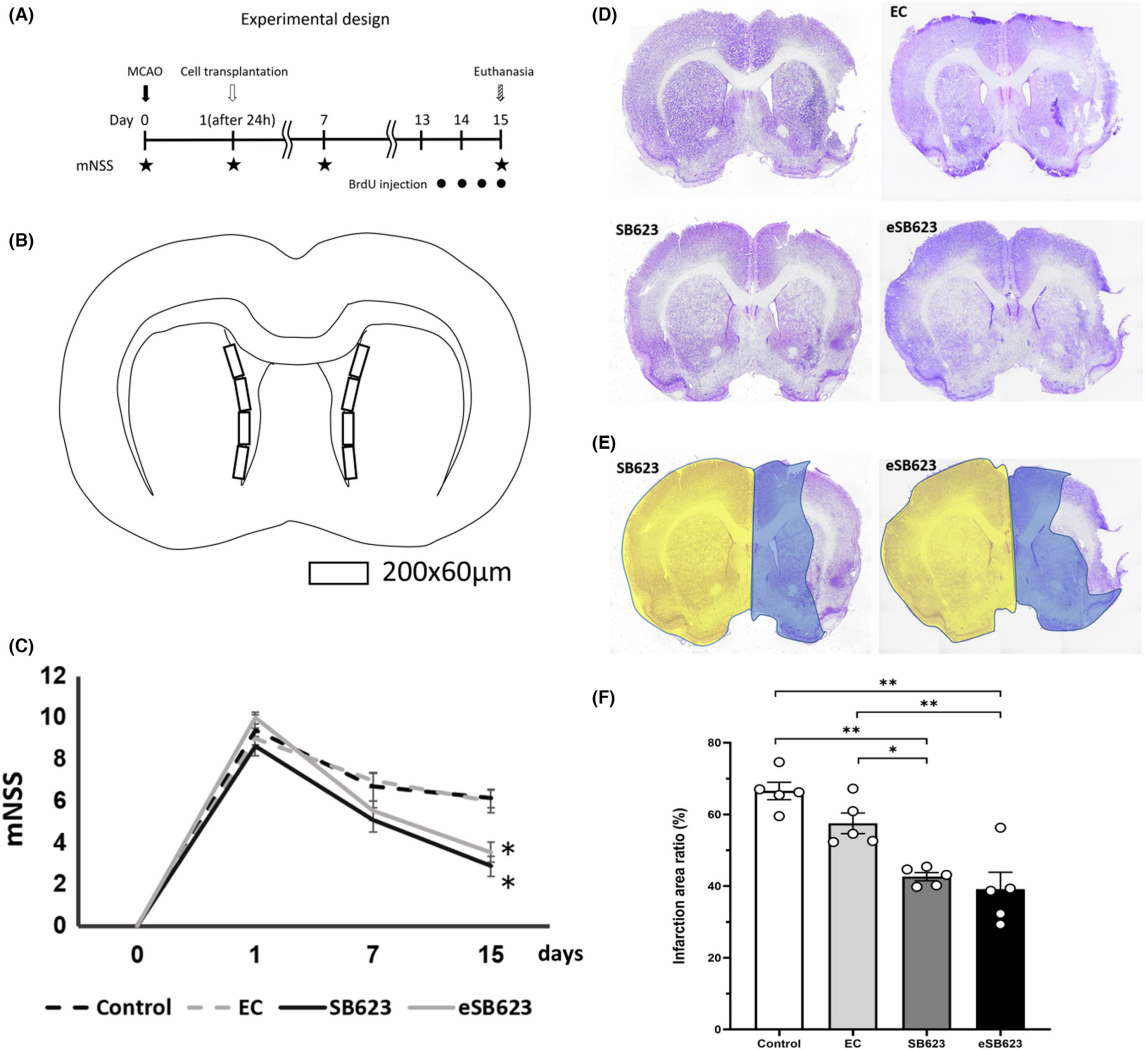
Experimental design, and evaluation of behavioral and infarction area. Experimental design for this study is shown (A). All rats underwent MCAO on day 0, and transplantation was performed on day 1. Modified neurological severity score was evaluated on day 0, 1, 7, and 15. All rats were euthanized on day 15. BrdU was administered to all rats at a concentration of 50 mg/kg body weight, with four consecutive intraperitoneal injections every 12 h from day 13 to 15. BrdU/Dcx double positive cells in the subventricular zone (SVZ) was evaluated like Figure (B). BrdU/Dcx double positive cells were counted bilaterally in four defined areas (200 × 60 μm) of the bilateral lateral ventricle wall. We counted 16 areas (4 areas × 2 sections × 2 sides) in each rat. Behavioral evaluation was shown (C). SB623 and eSB623 groups showed significantly better mNSS score than control and EC groups at day 15 (*n* = 7 in control group, *n* = 9 in EC group, *n* = 8 in SB623 group, and *n* = 9 in eSB623 group, the means ± SEM, *F*_(3, 29)_ = 11.33, *p < 0.05). On day 7, functional improvement was observed in the treatment group, but there was no significant change (*F*_(3, 29)_ = 2.391; eSB623 vs. control group: *p* = 0.425, eSB623 vs. EC group: *p* = 0.190, SB623 vs. control group: *p* = 0.165, SB623 vs. EC group: *p* = 0.053). Representative Nissl stains of all groups are shown (D). We calculated the infarction area ratio, defining LT, RT, and RI. LT is the area of the left hemisphere in mm^2^, RT is the area of the right hemisphere in mm^2^ and RI is the infarction area in mm^2^. Infarction area ratio = [LT − (RT − RI)] × 100 / LT (%). LT is yellow, RT is blue, and RI is uncolored (E). The results of the infarction area ratio are shown (F). The infarction area ratio on day 15 after MCAO was reduced significantly in SB623 and eSB623 groups compared to control and EC groups (*F*_(3, 16)_ = 17.64, each *n* = 5, the means ± SEM, *p < 0.05 **p < 0.01). BrdU, 5‐bromo‐2′‐deoxyuridine; Dcx, Doublecortin; EC, empty capsule; eSB623, encapsulated SB623; MCAO, middle cerebral artery occlusion; mNSS, modified neurological severity score.

The time course of this experiment is shown in Figure 1A.

### 2.2 Transient MCAO

We performed transient middle cerebral artery occlusion (MCAO). MCAO was induced according to the intraluminal suture method used in our previous studies.^25^, ^27^, ^28^, ^29^ Under general anesthesia (2% sevoflurane in 70% N₂O and 30% O₂), a 4–0 monofilament nylon suture with silicone‐coated tip (Xantopren L blue & ACTIVATOR Universal Liquid, Heraeus Kulzer GmbH & Co. KG) was inserted through an arteriotomy of the right external carotid artery into the origin of the right MCA. After MCAO for 90 min, the filament was withdrawn to restore the blood flow and the wound was sutured.

### 2.3 SB623 cells derivation and preparation

SB623 cells were provided by SanBio, Inc., and prepared in reference to previous reports. SB623 characterization were described previously.^11^, ^16^, ^17^, ^19^, ^30^, ^31^ In brief, bone marrow aspirates of healthy young adult human donors were transfected with an expression vector encoding NICD (amino acids 1703–2504), expanded, harvested, cryopreserved and stored in the vapor phase of liquid nitrogen. Frozen vials containing SB623 cells were placed into a 37°C water bath until completely thawed. Once thawed, they were immediately removed, and cells were transferred into a 15 ml conical centrifuge tube containing 10 ml of cold Dulbecco's phosphate‐buffered saline (PBS). The preparations were centrifuged at 1000 rpm (200 × g) in a swinging bucket rotor for 8 min at room temperature (RT) to form a pellet of cells. The supernatant was carefully removed and Dulbecco's modified Eagle's medium (DMEM; Sigma‐Aldrich, St. Louis, Missouri, United States), without serum or antibacterial agent, was added to yield a final cell concentration of about 8.0 × 10^4^ cells/μl. Cell counting was done to obtain the desired cell count and to check viability. After cell grafting, the remaining cells were checked and the number of viable cells was verified by trypan blue exclusion.^30^, ^31^ The results of cell concentration were in the range of 80,500 ± 25,400 cells/μl, with a viability range of 96.8 ± 2.29%.

### 2.4 Encapsulation

Encapsulation was performed as in our previous studies.^22^, ^23^, ^24^, ^25^ Sterile polymeric hollow fibers (15 cm in length) consisting of a semipermeable membrane (Amicon) were cut to 7 mm in length and used as capsules. These fibers were made of polysulfone (molecular cut‐off: 100 kDa), and the capsules had an inner diameter of 700 μm. Capsules (7 mm in length) were sealed at the distal end just before cell loading by applying photo‐curable cement. SB623 cells were prepared as a single‐cell suspension at a density of 8.0 × 10^4^ cells/μl resembling the same protocol for SB623 cell transplantation as described above. 5 μl of the cell solution was loaded into the proximal end of the hollow fibers. The access port was sealed with photo‐curable cement. The encapsulated SB623 cells were implanted within 1 h of encapsulation.

### 2.5 Cell transplantation

On day 1, cell transplantation was performed at 24 h after MCAO as in our previous studies.^22^, ^27^, ^29^ All rats received anesthesia with 0.3 mg/kg of medetomidine, 4.0 mg/kg of midazolam, and 5.0 mg/kg of butorphanol by intraperitoneal injection and placed in a stereotaxic instrument (Narishige, Japan). They underwent a midline head skin incision and a small hole drilled into their skull. SB623 cells (4.0 × 10^5^ cells/5 μl), encapsulated SB623 cells, or an empty capsule was unilaterally implanted into the right striatum. One capsule was implanted for each rat. They were transplanted in accordance with the following stereotactic coordinates^22^, ^24^: 1.0 mm anterior to the bregma, 3.0 mm lateral to the sagittal suture, and 5.0 mm ventral to the surface of the brain for targeting the right striatum. The tooth bar was set at 0.0 mm in all procedures.

### 2.6 Behavioral tests

Modified neurological severity score (mNSS) was performed on day 0, 1, 7, and 15. The mNSS assessed motor function, sensory disturbance, reflex, and balance. Neurological function was graded on a scale of 0 to 18 (normal score: 0, maximal score: 18).^32^, ^33^ Only rats showing 7–12 points of the mNSS at 24 h after reperfusion were included in this study.^27^ The final subject enrolment included control group (*n* = 7), empty capsule (EC) group (*n* = 9), SB623 group (*n* = 8), and encapsulated SB623 (eSB623) group (*n* = 9). The behavioral tests were performed by two investigators blinded to the treatments.

### 2.7 5‐bromo‐2′‐deoxyuridine injection

To label proliferating cells, 5‐bromo‐2′‐deoxyuridine (BrdU, NACALAI TESQUE INC.) was administered to all rats at a concentration of 50 mg/kg body weight, over four consecutive intraperitoneal injections every 12 h from day 13 to 15.^22^, ^23^

### 2.8 Histological analysis

Histological analysis was performed in all groups using randomly selected animals (*n* = 5 in each group). Nissl stain was performed to evaluate the infarction area.^27^, ^34^ On day 15 after MCAO, all rats were euthanized with an overdose of pentobarbital (100 mg/kg) and perfused through ascending aorta with 200 ml of cold PBS and 200 ml of 4% paraformaldehyde (PFA) in PBS. Brains were removed from rats and post‐fixed in the same fixative overnight at 4°C, and subsequently stored in 30% sucrose in PBS for 1 week. Coronal sections were cut at 30 μm thickness with a freezing microtome (−20°CC). These sections were mounted onto slides. In all groups, the infarction area ratio was measured at just anterior to the graft of the capsule using computerized image analysis (Image J; National Institutes of Health, Bethesda, USA). We evaluated the infarction area ratio by the following method: infarction area ratio = [LT – (RT – RI)] × 100 / LT (%), where LT is the area of the left hemisphere in mm^2^, RT is the area of the right hemisphere in mm^2^, and RI is the infarction area in mm^2^.^27^, ^35^, ^36^

BrdU/doublecortin (Dcx) double staining and quantification of BrdU/Dcx positive cells were performed to evaluate neurogenesis in the subventricular zone (SVZ) as in our previous studies.^22^, ^23^ All rats were euthanized, and brains were post‐fixed and cut into a coronal section at 30 μm thickness as described above. These free‐floating sections were incubated in 2 N HCl at 37°C for 20 min. This was followed by sodium borate incubation (pH 8.5) for 10 min. After washing three times with PBS, the sections were incubated for 24 h at 4°C with rat anti‐BrdU antibody (1:100, OBT0030G; Bio‐Rad), rabbit anti‐Dcx antibody (1:200, #4604; Cell Signaling Technology), 10% normal horse serum (Invitrogen) and 0.1% TritonX (NACALAI TESQUE INC.). After washing several times in PBS, the sections were incubated for 90 min with biotinylated anti‐rat secondary antibody (1:100, 712‐065‐153; Jackson Immunoresearch). Thereafter, the sections were washed three times in PBS and incubated for 1 h with Streptavidin Alexa‐488 (1:200, S11223; Invitrogen), goat anti‐rabbit IgG Cy3 (1:200, ab97075; Abcam), and 4,6‐diamidino‐2‐phenylindole (DAPI; 1:500, D3751; Thermo Fisher). Finally, the sections were washed three times in PBS and mounted on albumin‐coated glass slides. Immunoreactivities were visualized using a confocal laser scanning microscope LSM780 (Carl Zeiss) and corrected by ZEN Lite (Carl Zeiss) for the assessment of co‐localization of BrdU/Dcx‐positive cells.

Quantification of BrdU/Dcx positive cells in the subventricular zone (SVZ) was performed as previously described.^22^, ^37^, ^38^ BrdU/Dcx‐positive cells were counted bilaterally in four defined areas (200 × 60 μm) of the lateral ventricle wall. For cell counting, two sections per rats in the same position as the bregma were selected. In total, we counted 16 areas (4 areas × 2 sections × 2 sides) for the SVZ in each rat (Figure 1B). Each rat was considered an individual observation for statistical analysis.

### 2.9 Evaluation of the viability of encapsulated SB623 cells and the SB623 cells in the rats' brains

We evaluated the viability of encapsulated SB623 cells and SB623 cells transplanted into the brains of rats using two additional cohorts of animals. Transplantation of SB623 cells or encapsulated SB623 cells was performed in 15 and 12 rats, respectively. SB623 cells or encapsulated SB623 cells was transplanted using the same procedures as described above. Randomly selected rats from each group were euthanized on day 3, 7, and 14 for cell viability analyses. Anti‐STEM101/DAPI staining was performed as previously described.^39^ All rats were euthanized, and then the brains were post‐fixed and cut into a coronal section at 30 μm thickness as described above. Free‐floating sections were incubated at 4°C with anti‐STEM101 antibody (1:100, mouse anti‐human nuclei monoclonal antibody; Takara Bio, Inc.), 10% normal horse serum, and 0.1% TritonX (NACALAI TESQUE INC.). After several rinses, sections were incubated for 90 min with biotinylated anti‐rat secondary antibody (1:100, 712‐065‐153; Jackson Immunoresearch). Next, the sections were washed three times in PBS and incubated for 1 h in goat anti‐mouse IgG Alexa fluor 594 (Abcam) with 4,6‐diamidino‐2‐phenylindole (DAPI; 1:500, D3751; Thermo Fisher). The sections were washed three times in PBS and mounted on albumin‐coated glass slides. Immunoreactivities were visualized as described above, and we assessed the viable SB623 cells in the striatum.^41^ Subsequently, the viable cells in the striatum were counted in every six sectioned slices per rat and summed up.

Additionally, the viability of post‐implant encapsulated cells was evaluated on day 0, day 7, day 14, and day 28 after retrieval (*n* = 3 in each day). Encapsulated SB623 cells were retrieved after euthanasia. The encapsulated cells were fixed with 1 ml of 4% PFA overnight. Thereafter, the capsule was embedded in paraffin and cut into the section at 4 μm thickness with a microtome. Sections were mounted on glass slides and stained for hematoxylin and eosin.^23^, ^24^, ^40^, ^42^ Viable cells with nuclei were counted in five sections per capsule. Viable cells were calculated in each capsule, and the averages on day 0, 7, 14 and 28 were used for statistical analyses. In both groups, cell counting was also done to obtain the desired cell count and to check viability as described above immediately prior to transplantation. Both data were statistically analyzed with Mann–Whitney *U* test.

### 2.10 Statistical analyses

We analyzed data using IBM SPSS Statistics version 20.0 (IBM) and presented as the means ± standard error (SEM). All data were statistically evaluated using the Kolmogorov–Smirnov test to confirm for normal distribution. Because all data were not rejected the null hypothesis of normal distribution, we analyzed these results using one‐way analysis of variance (ANOVA) followed by Tukey's test. Statistical significance was preset at a *p*‐value <0.05.

## 3 RESULTS

### 3.1 SB623 and eSB623 treatments ameliorate stroke‐induced behavioral deficits

Modified neurological severity score was performed on day 0, 1, 7, and 15. Most of the rats of treatment groups showed recovery from hemiparalysis on day 15. ANOVA revealed significant treatment effects of SB623 and eSB623 groups in mNSS (Figure 1C). SB623 and eSB623 groups showed significantly better mNSS score than control and EC groups at day 15 (*F*_(3, 29)_ = 11.33, control group: 6.14 ± 0.46, EC group: 6.0 ± 0.53, SB623 group: 2.89 ± 0.48, eSB623 group: 3.56 ± 0.47; eSB623 vs control and EC groups: *p* < 0.05, SB623 vs. control and EC groups: *p* < 0.05). On day 7, a trend of functional improvement was observed in the treatment group, but SB623 and eSB623 groups did not reach statistically significant improvement in mNSS score compared to control and EC groups (*F*_(3, 29)_ = 2.391; eSB623 vs. control group: *p* = 0.425, eSB623 versus EC group: *p* = 0.190, SB623 vs control group: *p* = 0.165, SB623 vs. EC group: *p* = 0.053).

### 3.2 SB623 and eSB623 treatments reduce cerebral infarction

Nissl stain was performed on day15 after MCAO. In the treatment groups, Nissl stain showed reduction of infarction area compared to control and EC group (Figure 1D,E). ANOVA (Figure 1F) showed significant treatment effects on cerebral infarction as evidenced by significant reductions in Nissl‐stained infarcted areas in SB623 and eSB623 groups compared to control and EC groups (*F*_(3, 16)_ = 17.64, control group: 66.4 ± 2.30%, EC group: 57.5 ± 2.88%, SB623 group: 42.6 ± 1.14%, eSB623 group: 39.2 ± 4.68%; eSB623 vs. control and EC groups: *p* < 0.01, SB623 vs. control group: *p* < 0.01, SB623 vs. EC group: *p* < 0.05).

### 3.3 Implantation of encapsulated SB623 increases the number of BrdU/Dcx positive cells in the SVZ

**FIGURE 2:**
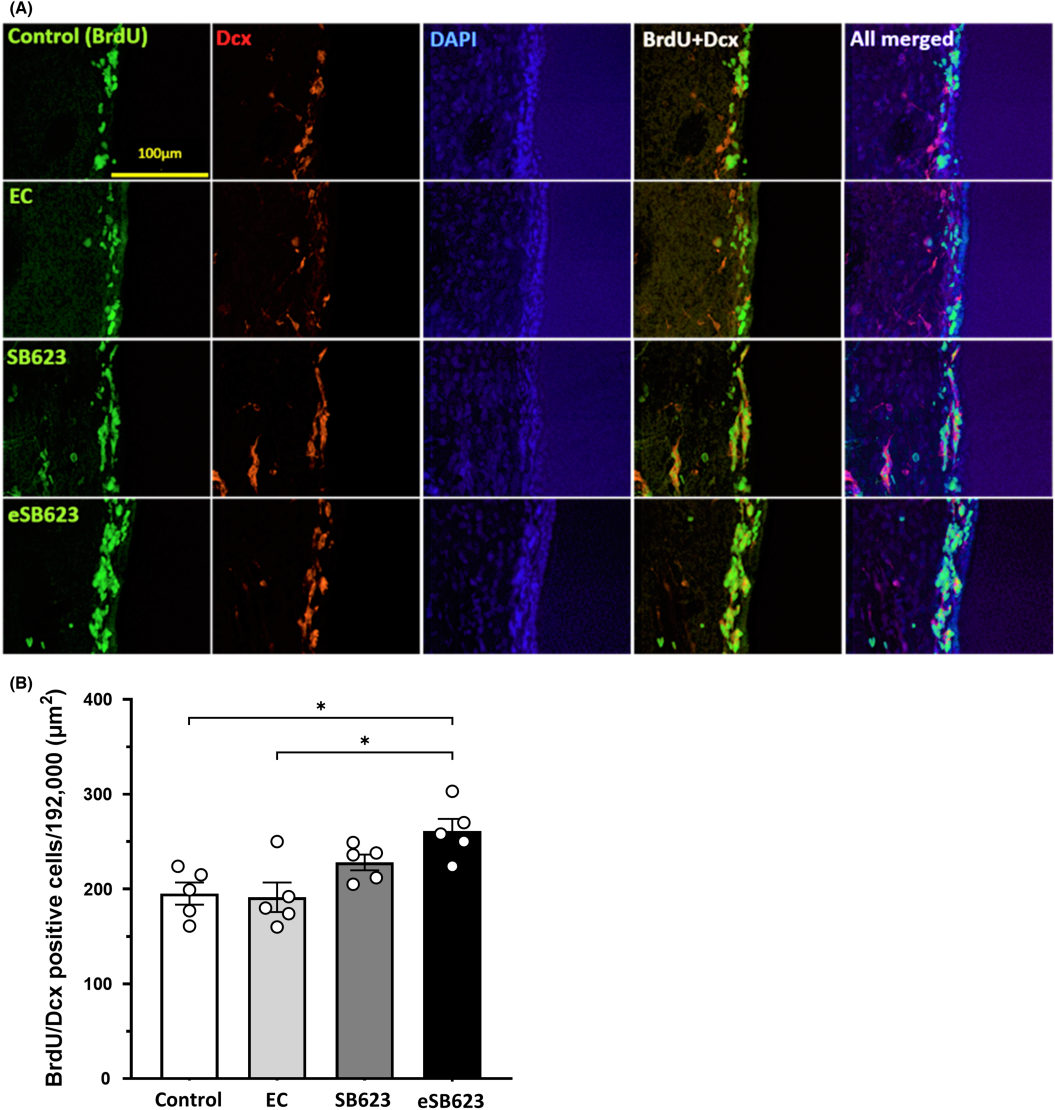
Neurogenesis in the SVZ. Immunostaining for BrdU (green), Dcx (red), and DAPI (blue) in the SVZ shows enhanced neurogenesis in the SVZ of eSB623 and SB623 groups (A). The number of BrdU/Dcx positive cells in the SVZ increased significantly in eSB623 group compared to control and EC groups (*F*_(3, 16)_ = 6.375, each *n* = 5, the means ± SEM, *p < 0.05). In SB623 group, the number of BrdU/Dcx positive cells in the SVZ increased compared to control and EC groups, but there was no significant statistical change (B) (vs. control group: *p* = 0.312; vs. EC group: *p* = 0.224). BrdU, 5‐bromo‐2′‐deoxyuridine; Dcx, Doublecortin; DAPI, 4,6‐diami‐dino‐2‐phenylindole; SVZ, subventricular zone; EC, empty capsule; eSB623, encapsulated SB623.

BrdU/Dcx double staining was performed on day 15. The fluorescent staining showed increase of neurogenesis in the SVZ (Figure 2A). ANOVA detected significant treatment effects on neurogenesis with the number of BrdU/Dcx positive cells in the bilateral SVZ significantly increased in eSB623 group compared to control and EC groups (*F*_(3, 16)_ = 6.375, control group: 195.2 ± 12.5, EC group: 191.2 ± 16.2, SB623 group: 228 ± 8.83, eSB623 group: 261 ± 13.1; *p* < 0.05, Figure 2B). Also in the contralateral SVZ, the number of BrdU/Dcx positive cells significantly increased in eSB623 group compared to control and EC groups (*F*_(3, 16)_ = 8.574, control group; 96.6 ± 5.52, EC group; 99.6 ± 8.29, SB623 group; 117.2 ± 8.92, eSB623 group; 143.6 ± 6.29; *p* < 0.05). In contrast, while there was a trend of increased number of BrdU/Dcx positive cells in the SVZ in SB623 group compared to control and EC groups, but the levels of neurogenesis between groups did not reach statistical significance (vs. control group: *p* = 0.312; vs. EC group: *p* = 0.224).

### 3.4 Significant correlations exist among mNSS score, infarction area ratio, and neurogenesis in the SVZ

**FIGURE 3:**
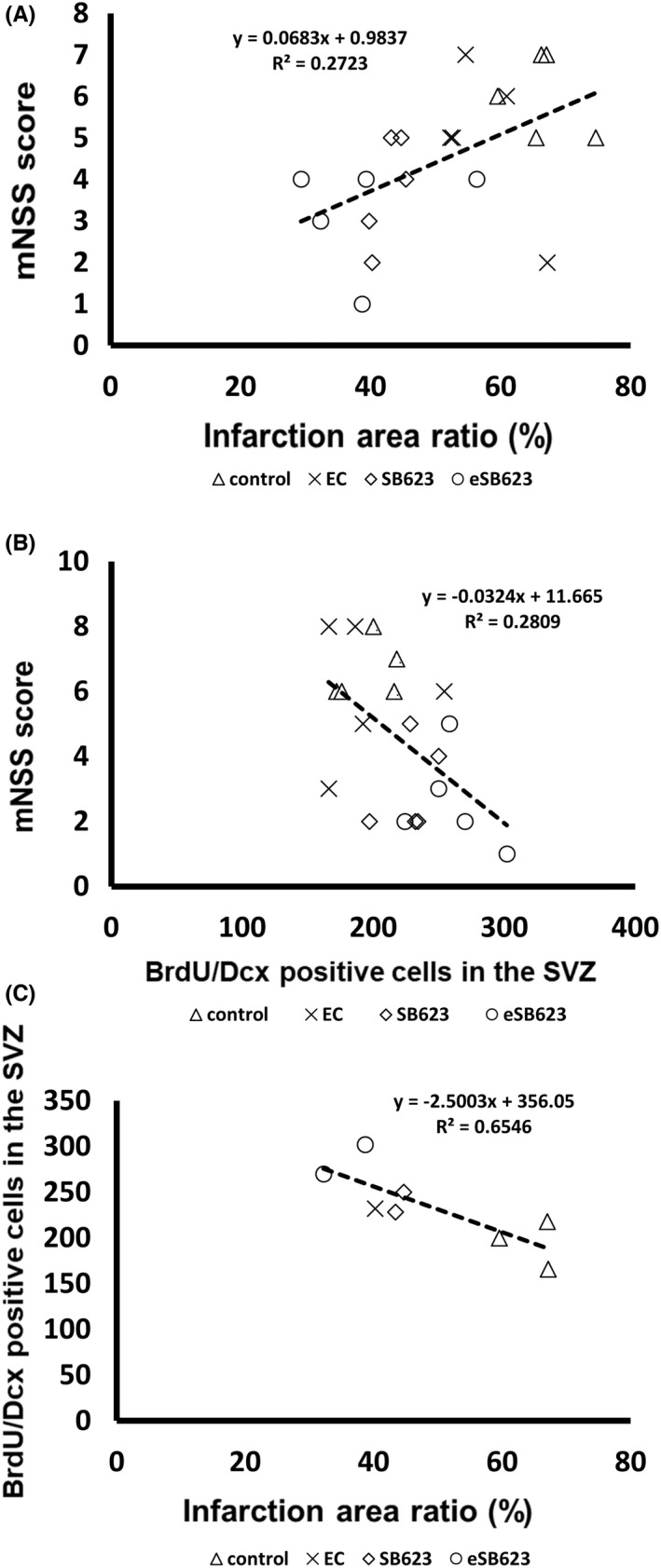
Correlational analyses of functional outcomes. There is a significant positive correlation between mNSS score and infarction area ratio (A) (*n* = 20, *r* ^2^: 0.5218, *p* < 0.05). A significant negative correlation between mNSS score and neurogenesis in the SVZ is shown (B) (*n* = 20, *r* ^2^: −0.5300, *p* < 0.05). A significant negative correlation between neurogenesis in the SVZ and infarction area ratio is shown (C) (*n* = 8, *r* ^2^: −0.8091, *p* < 0.05). mNSS: modified neurological severity score, BrdU, 5‐bromo‐2′‐deoxyuridine; Dcx, Doublecortin; DAPI, 4,6‐diami‐dino‐2‐phenylindole; EC, empty capsule; eSB623, encapsulated SB623.

Correlational analyses using Pearson correlation coefficient revealed a close interplay among mNSS score, infarction area ratio, and neurogenesis in the SVZ. First, a significant positive correlation between mNSS score and infarction area ratio was observed (*n* = 20, *r* ^2^: 0.5218, *p* < 0.05, Figure 3A). Second, a significant negative correlation was also detected between mNSS score and neurogenesis in the SVZ (*n* = 20, *r* ^2^: −0.5300, *p* < 0.05, Figure 3B). Third, there was a significant negative correlation between neurogenesis in the SVZ and infarction area ratio (*n* = 8, *r* ^2^: −0.8091, *p* < 0.05, Figure 3C).

### 3.5 Encapsulation prolongs cell survival of SB623 cells transplanted into the stroke brain

**FIGURE 4:**
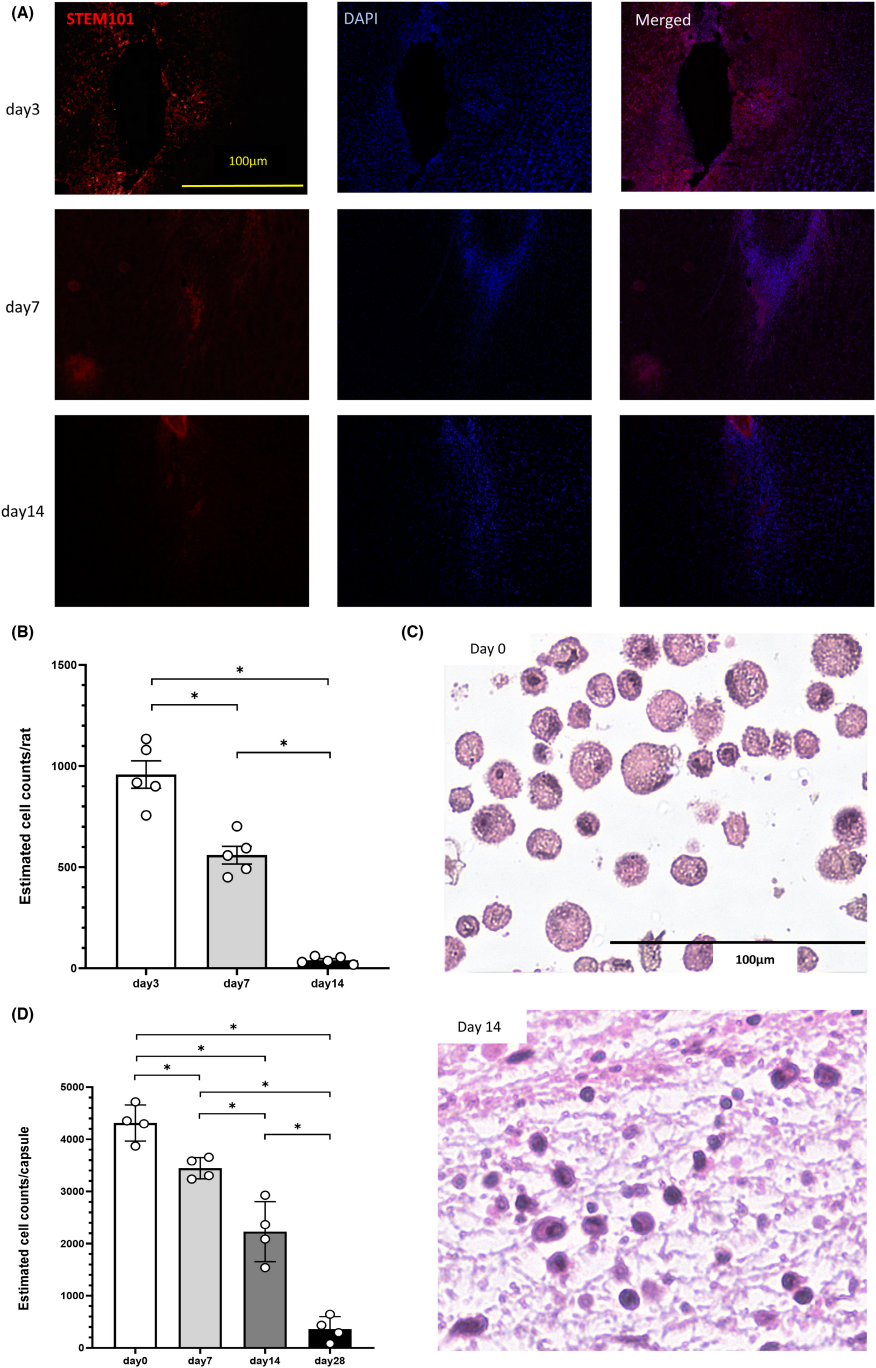
Viability of SB623 and eSB623 cells transplanted into the stroke brain. Representative images of immunostaining in each group are shown (A). The number of STEM101/DAPI positive cells decreased significantly on day 7 and day 14 compared to day 3 (B). (*F*_(2, 12)_ = 484.0, each *n* = 5, the means ± SEM, *p < 0.05). The hematoxylin and eosin staining of day 0 and day14 are shown (C). The encapsulated cells decreased significantly on day 14, but about 50% of day 0 was alive on day 14. On day 28, only 8% of the cells on day 0 survived (D). (*F*_(3, 12)_ = 84.1, each *n* = 4, the means ± SEM, *p < 0.05). Furthermore, significantly more encapsulated cells remained alive throughout the post‐transplant survival period compared to SB623. (day 7: *F*_(1, 7)_ = 782.9; *p* < 0.01, day 14: *F*_(1, 7)_ = 72.64; *p* < 0.01). DAPI, 4,6‐diami‐dino‐2‐phenylindole.

The viability of SB623 cells was evaluated on day 3, 7, 14, and the viability of encapsulated SB623 cells was evaluated on day 0, 7, 14, 28. A few encapsulated cells survived until day 28, and survival of SB623 cells on day14 was prolonged with encapsulation. The viability of SB623 cells was relatively high on day 3, but significantly decreased by day 7 (*F*_(2, 12)_ = 484.0, day 3: 957.6 ± 68.5, day 7: 559.8 ± 43.4, day 14: 39.6 ± 7.65; *p* < 0.05, Figure 4A,B). On the other hand, while there was also a decreasing trend in cell viability for eSB623, significantly more encapsulated cells remained alive throughout the post‐transplant survival period compared to SB623. (day 7: *F*_(1, 7)_ = 782.9; *p* < 0.01, day 14: *F*_(1, 7)_ = 72.64; *p* < 0.01). Cell counting and viability prior to transplantation had no significant difference between both groups (cell counting: *p* = 0.84, viability: *p* = 0.85, SB623 group: *n* = 15; 61,100 ± 32,900 cells/μ1, with a viability range of 92.1 ± 4.02%, eSB623 group: *n* = 12; 64,700 ± 34,300 cells/μ1, with a viability range of 91.9 ± 3.69%).

## 4 DISCUSSION

### 4.1 Encapsulated SB623 cells showed non‐inferiority in therapeutic effects comparable to SB623 cells transplanted directly into the stroke brain

This study initially compared the therapeutic effects of direct intracranial transplantation versus encapsulated cell transplantation of SB623 cells. Both treatment groups similarly improved behavioral score and reduced infarction area ratio compared to control and EC groups, but there was no significant difference between the two treatment groups. Encapsulated SB623 cells significantly enhanced neurogenesis in the SVZ compared to control and EC groups. Furthermore, encapsulated SB623 cells survived longer than SB623 cells in the rats' brains. Because the encapsulation prevented host cell‐grafted cell interaction and the formation of newly developed neural networks between the host and graft, which have been implicated in stem cell therapy‐mediated functional recovery,^22^, ^23^, ^24^ the present results support the by‐stander effects, i.e., secretion of neurotrophic factors, as the more dominant mechanism over cell replacement (e.g., cell–cell contact, neuronal differentiation, or brain circuitry reconstruction) mediating the therapeutic effects of transplanted SB623 cells.

### 4.2 Encapsulated SB623 cells survived longer than directly transplanted SB623 cells

Preclinical and clinical studies on transplantation of SB623 cells have demonstrated therapeutic effects in various neurological diseases like stroke, Parkinson's disease, and traumatic brain injury.^13^, ^17^, ^19^, ^30^, ^41^ The underlying mechanisms of these therapeutic effects are partially uncertain but implicate multi‐pronged regenerative mechanisms. Specifically, SB623 cells secrete factors that protect cells from hypoxic injury,^14^ support damaged cells,^15^, ^16^, ^43^ promote angiogenesis,^15^, ^16^ exert anti‐inflammatory effects,^16^ afford immunosuppressive effects,^16^, ^43^ secrete extracellular matrix proteins promoting neural cell growth,^12^ enhance neural stem cell migration and differentiation,^16^ and provide a biobridge of extracellular matrix metalloproteinases.^45,^ BMSCs, including SB623 cells, have been demonstrated to integrate or differentiate into injured neural cells and secrete supportive factors.^7^ Because SB623 cells survive for at most 1 month in xenogeneic rat's brains, their therapeutic effects may be achieved by secretion of supportive factors rather than cell replacement through neural integration or differentiation.^41^, ^44^ In our previous studies, we examined intracranial transplantation of encapsulated cell lines including BMSCs into a stroke or non‐stroke model of rats with consequent demonstration that the encapsulation enhanced neurological recovery.^22^, ^23^, ^24^, ^25^ With encapsulation, cells are protected from host immunoreaction and they survive longer than unencapsulated cells in vivo.^22^, ^45^ In this study, we revealed that encapsulated SB623 cells survived longer than unencapsulated SB623 cells. We postulate that encapsulated SB623 cells secreted larger amounts of supportive neurotrophic factors over an extended period of time, leading to recovery of the behavioral score, reduction in infarct area, and enhancement of neurogenesis in the SVZ. Interestingly, the viability of encapsulated cells at day 0 was not so high, likely due to the SB623 thawing protocol immediately preceding the cell transplantation that did not allow an ample time for the cells to recover from such harsh grafting procedure. Notwithstanding, the cell viability of encapsulated cells appears to robust at later time points post‐transplantation compared to the unencapsulated cells. The present protocol for SB623 cell thawing preparation and transplantation simulated the time course for clinical application of SB623 in the stroke setting.

### 4.3 Effects of xenogeneic transplantation

In this study, the human SB623 cell donor and the rat transplant recipient catered to a xenogeneic transplantation approach. The host's immune response poses as a major problem in any xenogeneic cell transplantation procedure. However, there is no detectable significant difference in the therapeutic outcomes between allogeneic intracranial transplantation of rat NICD‐transfected BMSCs and xenogeneic transplantation of human SB623 cells in chronic rat stroke model.^41^ Furthermore, SB623 cells display immunosuppressive potency comparable to untransfected human BMSCs. In addition, intracranial transplantation of human BMSCs into the ischemic stroke rats even without immunosuppression reduces infarct area and promotes neurogenesis in the SVZ.^46^, ^47^ Altogether these studies suggest that the minimal contribution of the host's immune response to either xenogeneic transplant model involving human SB623 cells or allogeneic approach employing rat BMSCs. Accordingly, the use of immunosuppression with the xenogeneic cells, as in the present study, may not be required for the envisioned clinical transplantation of SB623 cells. Coupled with the added encapsulation strategy, which should further dampen the immune response of the host to xenogeneic transplantation, it is likely that immune‐related graft‐versus‐host complications factored in the present study.

### 4.4 Neurotrophic factors secretion of encapsulated SB623 cells

The current study showed good viability of the encapsulated SB623 cells even up to day 14 post‐transplantation. Although we did not analyze here growth factor secretion, previous reports showed that secretion of MCP‐1, FGF‐1, and FGF‐2 was enhanced in SB623 cells compared to non‐transfected human BMSCs.^14^, ^15^, ^16^ MCP‐1 induces migration of neuroblasts from the SVZ to infarct region in rodents,^48^ while FGF‐1 and FGF‐2 play an important role in the growth of neural cells.^14^ Similar robust and extended secretion of neurotrophic factors, such as MCP‐1, FGF‐1, FGF‐2, likely complemented the encapsulation of SB623 cells in this study.

### 4.5 Study limitations

The observation that encapsulated SB623 cells promoted neurogenesis and increased grafted cell viability but did not enhance functional recovery compared to unencapsulated SB623 cells might have been masked by the limited study period. In our previous study, we monitored the therapeutic effects of encapsulated cells for half a year.^45^ Here, we examined encapsulated cells for only 2 weeks post‐transplantation. A long‐term survival period post‐transplantation period may reveal improved functional outcomes and histological proof of tissue regeneration with mature neurons by encapsulated SB623 cell. In addition, as noted above, we did not evaluate the secretion of growth factors between encapsulated and unencapsulated SB623 cells. The identification of distinct neurotrophic factors secreted by SB623 may reveal specific regenerative pathways that mediate the cells' therapeutic effects. Recently, sex differences in brain blood vessels, metabolism, and stroke outcomes have been discussed in clinical settings and basic research.^49^, ^50^ In our study, only male rats were used because of the limited number of animals for animal protection and the uniform results after MCAO. In the future, we need to keep sex differences in mind to perform stroke research.

## 5 CONCLUSION

We demonstrated SB623 cells with or without encapsulation exerted therapeutic effects on the acute ischemic stroke rats. Encapsulated SB623 cells enhanced neurogenesis and survived longer than unencapsulated SB623 cells in the stroke brain. Despite the absence of enhanced functional recovery in stroke rats that received the encapsulated SB623 cells compared to those with unencapsulated cells, the encapsulation created a barrier preventing host‐graft cell‐to‐cell contact and innervation, suggesting that the secretory function of SB623 serves as the dominant mechanism mediating the therapeutic effects of SB623 cells in acute stroke model. Further studies with long‐term post‐transplantation period might reveal the functional recovery or reduction of infarct area in encapsulated SB623 transplantation group through the prolonged secretion of trophic factors with enhanced neurogenesis, compared to direct transplantation of SB623.

## AUTHOR CONTRIBUTION

S.K.: conception and design, data collection, data analysis and interpretation, manuscript writing and final approval; T.Y.: conception and design, manuscript revision, final approval of manuscript; K.K.: conception and design, manuscript revision; T.N., C.S., S.Y.: data collection, manuscript revision; Y.O.: data collection; K.H.: data collection, assembly of data; Y.T.: data analysis and interpretation; M.U.: data collection, data analysis and interpretation; T.S.: assembly of data; M.K.: data collection, data analysis and interpretation, manuscript revision; CV.B.: manuscript revision, data interpretation; D.I.: manuscript revision, final approval of manuscript.

## CONFLICT OF INTEREST

The authors declare that there is no conflict of interest other than below. Cesar Borlongan is an Editorial Board member of CNS Neuroscience and Therapeutics and a co‐author of this article. To minimize bias, they were excluded from all editorial decision‐making related to the acceptance of this article for publication.

## Data Availability

The data that support the findings of this study are available from the corresponding author upon reasonable request.

## References

[1] Chen P , Gao S , Wang Y , Xu A , Li Y , Wang D . Classifying ischemic stroke, from TOAST to CISS. CNS Neurosci Ther. 2012;18(6):452‐456. 22268862 10.1111/j.1755-5949.2011.00292.x PMC6493455

[2] Vu Q , Xie K , Eckert M , Zhao W , Cramer SC . Meta‐analysis of preclinical studies of mesenchymal stromal cells for ischemic stroke. Neurology. 2014;82(14):1277‐1286. 24610327 10.1212/WNL.0000000000000278 PMC4001201

[3] Hao L , Zou Z , Tian H , et al. Stem cell‐based therapies for ischemic stroke. Biomed Res Int. 2014;468748. 10.1155/2014/468748 PMC3955655 24719869

[4] Shinozuka K , Dailey T , Tajiri N , Ishikawa H , Kaneko Y , Borlongan C . Stem cell transplantation for neuroprotection in stroke. Brain Sci. 2013;3(1):239‐261. 24147217 10.3390/brainsci3010239 PMC3800120

[5] Borlongan C . Concise review: stem cell therapy for stroke patients: are we there yet? Stem Cells Transl Med. 2019;8(9):983‐988. 31099181 10.1002/sctm.19-0076 PMC6708064

[6] Zheng H , Zhang B , Chhatbar P , et al. Mesenchymal stem cell therapy in stroke: a systematic review of literature in pre‐clinical and clinical research. Cell Transplant. 2018;27(12):1723‐1730. 30343609 10.1177/0963689718806846 PMC6300779

[7] Stonesifer C , Corey S , Ghanekar S , Diamandis Z , Acosta SA , Borlongan CV . Stem cell therapy for abrogating stroke‐induced neuroinflammation and relevant secondary cell death mechanisms. Prog Neurobiol. 2017;158:94‐131. 28743464 10.1016/j.pneurobio.2017.07.004 PMC5671910

[8] Ning G , Song W , Xu H , Zhu R , Wu Q . Bone marrow mesenchymal stem cells stimulated with low‐intensity pulsed ultrasound: Better choice of transplantation treatment for spinal cord injury: treatment for SCI by LIPUS‐BMSCs transplantation. CNS Neurosci Ther. 2019;25(4):496‐508. 30294904 10.1111/cns.13071 PMC6488928

[9] Wang L , Lin Z , Zhang H , Shao B . Timing and dose regimens of marrow mesenchymal stem cell transplantation affect the outcomes and neuroinflammatory response after ischemic stroke. CNS Neurosci Ther. 2014;20(4):317‐326. 24393245 10.1111/cns.12216 PMC6493061

[10] Chen J , Li Y , Katakowski M , et al. Intravenous bone marrow stromal cell therapy reduces apoptosis and promotes endogenous cell proliferation after stroke in female rat. J Neurosci Res. 2003;73(6):778‐786. 12949903 10.1002/jnr.10691

[11] Dezawa M , Kanno H , Hoshino M , et al. Specific induction of neuronal cells from bone marrow stromal cells and application for autologous transplantation [Research Support, Non‐U S Gov't]. J Clin Invest. 2004;113(12):1701‐1710. 15199405 10.1172/JCI20935 PMC420509

[12] Aizman I , Tate CC , McGrogan M , Case CC . Extracellular matrix produced by bone marrow stromal cells and by their derivative, SB623 cells, supports neural cell growth. J Neurosci Res. 2009;87(14):3198‐3206. 19530164 10.1002/jnr.22146

[13] Mimura T , Dezawa M , Kanno H , Yamamoto I . Behavioral and histological evaluation of a focal cerebral infarction rat model transplanted with neurons induced from bone marrow stromal cells. J Neuropathol Exp Neurol. 2005;64(12):1108‐1117. 16319721 10.1097/01.jnen.0000190068.03009.b5

[14] Aizman I , Tirumalashetty BJ , McGrogan M , Case CC . Comparison of the neuropoietic activity of gene‐modified versus parental mesenchymal stromal cells and the identification of soluble and extracellular matrix‐related neuropoietic mediators. Stem Cell Res Ther. 2014;5(1):29. 24572070 10.1186/scrt418 PMC4055059

[15] Dao M , Tate CC , McGrogan M , Case CC . Comparing the angiogenic potency of naive marrow stromal cells and Notch‐transfected marrow stromal cells [Comparative Study]. J Transl Med. 2013;11(81). 10.1186/1479-5876-11-81 PMC3615967 23531336

[16] Tate CC , Fonck C , McGrogan M , Case CC . Human mesenchymal stromal cells and their derivative, SB623 cells, rescue neural cells via trophic support following in vitro ischemia. Cell Transplant. 2010;19(8):973‐984. 20350349 10.3727/096368910X494885

[17] Kawabori M , Weintraub A , Imai H , et al. Cell Therapy for Chronic TBI: Interim Analysis of the Randomized Controlled STEMTRA Trial. Neurology. 2021;96(8):e1202‐e1204. 10.1212/WNL.0000000000011450 PMC8055341 33397772

[18] Steinberg GK , Kondziolka D , Wechsler LR , et al. Clinical outcomes of transplanted modified bone marrow‐derived mesenchymal stem cells in stroke: a phase 1/2a study [Clinical Trial, Phase I Clinical Trial, Phase II Research Support, Non‐U S Gov't]. Stroke. 2016;47(7):1817‐1824. 27256670 10.1161/STROKEAHA.116.012995 PMC5828512

[19] Steinberg GK , Kondziolka D , Wechsler L , et al. Two‐year safety and clinical outcomes in chronic ischemic stroke patients after implantation of modified bone marrow‐derived mesenchymal stem cells (SB623): a phase 1/2a study. J Neurosurg. 2018;131(5):1462‐1472. 10.3171/2018.5.JNS173147 30497166

[20] Kawabori M , Kuroda S , Sugiyama T , et al. Intracerebral, but not intravenous, transplantation of bone marrow stromal cells enhances functional recovery in rat cerebral infarct: an optical imaging study. Neuropathology. 2012;32(3):217‐226. 22007875 10.1111/j.1440-1789.2011.01260.x

[21] Saraf J , Sarmah D , Vats K , et al. Intra‐arterial stem cell therapy modulates neuronal calcineurin and confers neuroprotection after ischemic stroke. Int J Neurosci. 2019;129(10):1039‐1044. 31203689 10.1080/00207454.2019.1633315

[22] Kin K , Yasuhara T , Kameda M , et al. Cell encapsulation enhances antidepressant effect of the mesenchymal stem cells and counteracts depressive‐like behavior of treatment‐resistant depressed rats. Mol Psychiatry. 2020;25:1202‐1214. 30108315 10.1038/s41380-018-0208-0

[23] Kuramoto S , Yasuhara T , Agari T , et al. BDNF‐secreting capsule exerts neuroprotective effects on epilepsy model of rats. Brain Res. 2011;1368(12):281‐289. 20971090 10.1016/j.brainres.2010.10.054

[24] Fujiwara K , Date I , Shingo T , et al. Reduction of infarct volume and apoptosis by grafting of encapsulated basic fibroblast growth factor‐secreting cells in a model of middle cerebral artery occlusion in rats. J Neurosurg. 2003;99(6):1053‐1062. 14705734 10.3171/jns.2003.99.6.1053

[25] Yano A , Shingo T , Takeuchi A , et al. Encapsulated vascular endothelial growth factor‐secreting cell grafts have neuroprotective and angiogenic effects on focal cerebral ischemia. J Neurosurg. 2005;103(1):104‐114. 16121981 10.3171/jns.2005.103.1.0104

[26] Yasuhara T , Shingo T , Kobayashi K , et al. Neuroprotective effects of vascular endothelial growth factor (VEGF) upon dopaminergic neurons in a rat model of Parkinson's disease. Eur J Neurosci. 2004;19:1494‐1504. 15066146 10.1111/j.1460-9568.2004.03254.x

[27] Morimoto J , Yasuhara T , Kameda M , et al. Electrical stimulation enhances migratory ability of transplanted bone marrow stromal cells in a rodent ischemic stroke model. Cell Physiol Biochem. 2018;46(1):57‐68. 29587284 10.1159/000488409

[28] Kidani N , Hishikawa T , Hiramatsu M , et al. Cerebellar blood flow and gene expression in crossed cerebellar diaschisis after transient middle cerebral artery occlusion in rats. Int J Mol Sci. 2020;21(11):4137. 10.3390/ijms21114137 PMC7312675 32531947

[29] Toyoshima A , Yasuhara T , Kameda M , et al. Intra‐arterial transplantation of allogeneic mesenchymal stem cells mounts neuroprotective effects in a transient ischemic stroke model in rats: analyses of therapeutic time window and its mechanisms. PLoS One. 2015;10(6):e0127302. 26075717 10.1371/journal.pone.0127302 PMC4468176

[30] Glavaski‐Joksimovic A , Virag T , Mangatu TA , McGrogan M , Wang XS , Bohn MC . Glial cell line‐derived neurotrophic factor‐secreting genetically modified human bone marrow‐derived mesenchymal stem cells promote recovery in a rat model of Parkinson's disease [Research Support, Non‐U S Gov't]. J Neurosci Res. 2010;88(12):2669‐2681. 20544825 10.1002/jnr.22435

[31] Tate CC , Chou VP , Campos C , et al. Mesenchymal stromal SB623 cell implantation mitigates nigrostriatal dopaminergic damage in a mouse model of Parkinson's disease. J Tissue Eng Regen Med. 2017;11(6):1835‐1843. 26440859 10.1002/term.2081

[32] Chen J , Sanberg P , Li Y , et al. Intravenous administration of human umbilical cord blood reduces behavioral deficits after stroke in rats. Stroke. 2001;32(11):2682‐2688. 11692034 10.1161/hs1101.098367

[33] Chen Y , Constantini S , Trembovler V , et al. An experimental model of closed head injury in mice: pathophysiology, histopathology, and cognitive deficits. J Neurotrauma. 1996;13(10):557‐568. 8915907 10.1089/neu.1996.13.557

[34] Popp A , Jaenisch N , Witte O , et al. Identification of ischemic regions in a rat model of stroke. PLoS one. 2009;4(3):e4764. 19274095 10.1371/journal.pone.0004764 PMC2652027

[35] Kameda M , Shingo T , Takahashi K , et al. Adult neural stem and progenitor cells modified to secrete GDNF can protect, migrate and integrate after intracerebral transplantation in rats with transient forebrain ischemia. Eur J Neurosci. 2007;26(6):1462‐1478. 17880388 10.1111/j.1460-9568.2007.05776.x

[36] Neumann‐Haefelin T , Kastrup A , de Crespigny A , et al. Serial MRI after transient focal cerebral ischemia in rats: dynamics of tissue injury, blood‐brain barrier damage, and edema formation. Stroke. 2000;31(8):1965‐1972. 10926965 10.1161/01.str.31.8.1965

[37] Yasuhara T , Hara K , Maki M , et al. Lack of exercise, via hindlimb suspension, impedes endogenous neurogenesis. Neuroscience. 2007;149(1):182‐191. 17869433 10.1016/j.neuroscience.2007.07.045

[38] Baldauf K , Reymann K . Influence of EGF/bFGF treatment on proliferation, early neurogenesis and infarct volume after transient focal ischemia. Brain Res. 2005;1056(2):158‐167. 16125154 10.1016/j.brainres.2005.07.035

[39] Salazar D , Uchida N , Hamers F , et al. Human neural stem cells differentiate and promote locomotor recovery in an early chronic spinal cord injury NOD‐scid mouse model. PLoS one. 2010;5(8):e12272. 20806064 10.1371/journal.pone.0012272 PMC2923623

[40] Ono S , Date I , Nakajima M , et al. Three‐dimensional analysis of vasospastic major cerebral arteries in rats with the corrosion cast technique. Stroke. 1997;28(8):1631‐1637. 9259761 10.1161/01.str.28.8.1631

[41] Yasuhara T , Matsukawa N , Hara K , et al. Notch‐induced rat and human bone marrow stromal cell grafts reduce ischemic cell loss and ameliorate behavioral deficits in chronic stroke animals. Stem Cells Dev. 2009;18(10):1501‐1514. 19301956 10.1089/scd.2009.0011

[42] Date I , Shingo T , Ohmoto T , Emerich DF . Long‐term enhanced chromaffin cell survival and behavioral recovery in hemiparkinsonian rats with co‐grafted polymer‐encapsulated human NGF‐secreting cells. Exp Neurol. 1997;147(1):10‐17. 9294398 10.1006/exnr.1997.6579

[43] Dao MA , Tate CC , Aizman I , McGrogan M , Case CC . Comparing the immunosuppressive potency of naive marrow stromal cells and Notch‐transfected marrow stromal cells. J Neuroinflammation. 2011;8(133):1742‐2094. 10.1186/1742-2094-8-133 PMC3228829 21982515

[44] Tajiri N , Kaneko Y , Shinozuka K , et al. Stem cell recruitment of newly formed host cells via a successful seduction? Filling the gap between neurogenic niche and injured brain site. PLoS One. 2013;8:e74857. 24023965 10.1371/journal.pone.0074857 PMC3762783

[45] Yoshida H , Date I , Shingo T , et al. Stereotactic transplantation of a dopamine‐producing capsule into the striatum for treatment of Parkinson disease: a preclinical primate study. J Neurosurg. 2003;98(4):874‐881. 12691415 10.3171/jns.2003.98.4.0874

[46] Jeong CH , Kim SM , Lim JY , Ryu CH , Jun JA , Jeun SS . Mesenchymal stem cells expressing brain‐derived neurotrophic factor enhance endogenous neurogenesis in an ischemic stroke model [in eng]. Biomed Res Int. 2014;129145:1‐10. 10.1155/2014/129145 PMC3933216 24672780

[47] Bao X , Wei J , Feng M , et al. Transplantation of human bone marrow‐derived mesenchymal stem cells promotes behavioral recovery and endogenous neurogenesis after cerebral ischemia in rats. Brain Res. 2011;1367:103‐113. 20977892 10.1016/j.brainres.2010.10.063

[48] Yan YP , Sailor K , Lang B , et al. Monocyte chemoattractant protein‐1 plays a critical role in neuroblast migration after focal cerebral ischemia. J Cereb Blood Flow Metab. 2007;27(6):1213‐1224. 17191078 10.1038/sj.jcbfm.9600432

[49] Chandra PK , Cikic S , Baddoo MC , et al. Transcriptome analysis reveals sexual disparities in gene expression in rat brain microvessels. J Cereb Blood Flow Metab. 2021;41(9):2311‐2328. 33715494 10.1177/0271678X21999553 PMC8392780

[50] Bushnell CD , Chatuvredi S , Gage KR , et al. Sex differences in stroke: challenges and opportunities. J Cereb Blood Flow Metab. 2018;38(12):2179‐2191. 30114967 10.1177/0271678X18793324 PMC6282222

